# Simultaneous Analysis of Iridoid Glycosides and Anthraquinones in *Morinda officinalis* Using UPLC-QqQ-MS/MS and UPLC-Q/TOF-MS^E^

**DOI:** 10.3390/molecules23051070

**Published:** 2018-05-03

**Authors:** Xiangsheng Zhao, Jianhe Wei, Meihua Yang

**Affiliations:** 1 Hainan Branch Institute of Medicinal Plant Development, Chinese Academy of Medical Sciences & Peking Union Medical College, Haikou 571100, China; xiangshengzhao@hotmail.com (X.Z.); wjianh@263.net (J.W.); 2 Institute of Medicinal Plant Development, Chinese Academy of Medical Sciences & Peking Union Medical College, Beijing 100193, China

**Keywords:** *Morinda officinalis*, iridoid glycosides, anthraquinones, fragmentation behaviors, LC-MS/MS

## Abstract

*Morinda officinalis* is an important herbal medicine and functional food, and its main constituents include anthraquinone and iridoid glycosides. Quantification of the main compounds is a necessary step to understand the quality and therapeutic properties of *M. officinalis*, but this has not yet been performed based on liquid chromatography/tandem mass spectrometry (LC-MS/MS). Analytes were extracted from *M. officinalis* by reflux method. Ultrahigh-performance liquid chromatography coupled with a triple quadrupole mass spectrometry (UPLC-QqQ-MS) using multiple reaction monitoring (MRM) mode was applied for quantification. Fragmentation pathways of deacetyl asperulosidic acid and rubiadin were investigated based on UPLC with quadrupole time-of-flight tandem mass spectrometry (Q/TOF-MS) in the MS^E^ centroid mode. The method showed a good linearity over a wide concentration range (R^2^ ≥ 0.9930). The limits of quantification of six compounds ranged from 2.6 to 27.57 ng/mL. The intra- and inter-day precisions of the investigated components exhibited an RSD within 4.5% with mean recovery rates of 95.32–99.86%. Contents of selected compounds in *M. officinalis* varied significantly depending on region. The fragmentation pathway of deacetyl asperulosidic and rubiadin was proposed. A selective and sensitive method was developed for determining six target compounds in *M. officinalis* by UPLC-MS/MS. Furthermore, the proposed method will be helpful for quality control and identification main compounds of *M. officinalis*.

## 1. Introduction

*Morinda officinalis* F.C. How (Rubiaceae) mainly grows in tropical and subtropical regions, such as Guangdong, Hainan, Guangxi, and Fujian provinces of China [1]. Its roots—an important traditional Chinese medicine (TCM) and functional food—have been widely used for the treatment of sexual impotence, spermatorrhea, irregular menstruation, and female infertility for more than 2000 years [2,3]. To date, pharmacological studies have revealed that *M. officinalis* exhibits antiosteoporosis, antifatigue, antioxidative, antidepressant, and hypoglycemic activities [4,5,6,7]. Iridoid glycosides, anthraquinones, polysaccharides, and oligosaccharides are the main bioactive constituents of *M. officinalis* [8,9]. In Chinese Pharmacopoeia (2015 edition), nystose assay using high-performance liquid chromatography (HPLC) and thin layer chromatography (TLC) identification was performed for quality control of *M. officinalis* [3]. As is known, herbal medicines exert their curative effects through multiple components on multiple targets [10]. Despite the beneficial effects and biological activities of anthraquinones and iridoids [4,11,12], few reports on their simultaneous identification and quantitation are available.

Currently, several analytical methods based on HPLC coupled to ultraviolet detection (UV) [13,14,15,16,17] or mass spectrometry (MS) (Li et al., 2016) have been reported for the determination of anthraquinones or iridoid glycosides in *M. officinalis*. However, these methods suffered from low resolution or long run-time, with few of these investigations being conducted for the simultaneous determination of multicomponents in this herbal medicine [18]. Therefore, development of a reliable approach to quantify multiple bioactive constituents for the comprehensive quality control of *M. officinalis* is of great importance. Ultrahigh-performance liquid chromatography–triple quadrupole mass spectrometry (UPLC-QqQ-MS/MS) method in multiple reaction monitoring (MRM) mode has drawn much attention for the analysis of active compounds in herbal medicines due to its high speed, improved sensitivity and specificity, and superior accuracy [19,20]. Furthermore, it is a powerful approach to solve the problems mentioned above, with added benefits of short run-times and less solvent consumption. In addition, due to its high resolution, sensitivity, and accuracy, UPLC-Q/TOF-MS/MS has become a dominant tool to analyze the chemical components of TCM. Moreover, it can also provide isotopic abundances and the elemental composition of fragment ions, which are greatly valuable to the structural inference of unknown ingredients [21,22]. 

**Figure 1:**
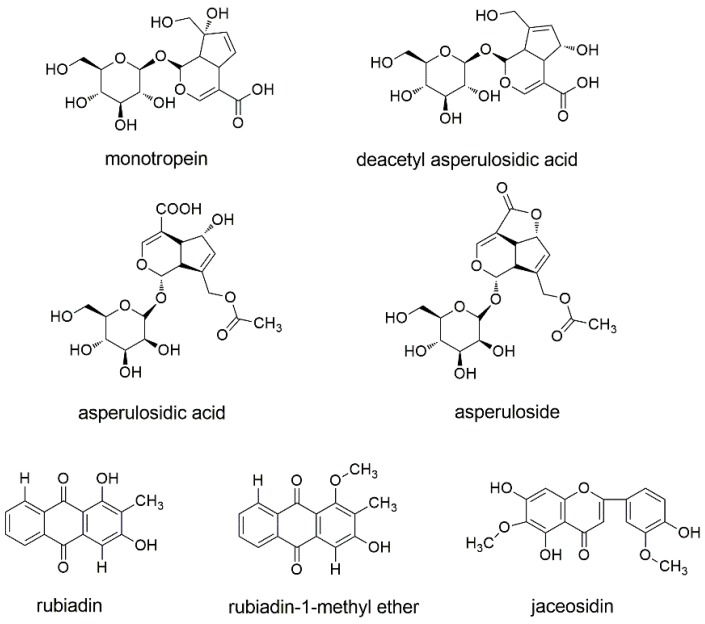
Chemical structures of the six compounds and jaceosidin (internal standard, IS).

Hence, in this work, we established a UPLC-QqQ-MS/MS method in the MRM mode for determining six active components (monotropein, deacetyl asperulosidic acid, asperulosidic acid, asperuloside, rubiadin-1-methyl ether, and rubiadin, Figure 1) in *M. officinalis* collected from different origins in China. Then, UPLC-Q/TOF-MS/MS was employed to further confirm the anthraquinones and iridoid glycosides in the methanolic extract of *M. officinalis*. The fragmentation behavior of iridoid glycosides and anthraquinones was explored in negative mode. This study served as the first example of comprehensive quality control and knowledge expansion for the quantification and identification of multiple compounds in *M. officinalis*.

## 2. Results and Discussion

### 2.1. Optimization of UPLC-QqQ-MS/MS Conditions

**Figure 2:**
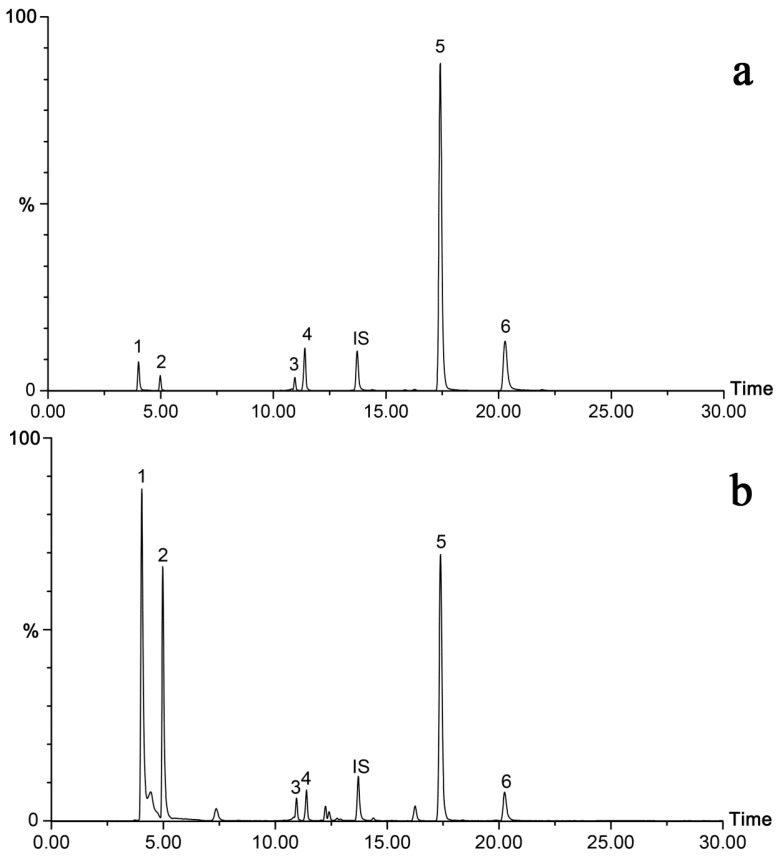
UPLC-MS/MS multiple reaction mode (MRM) chromatograms of (**a**) mixed standards and (**b**) *M. officinalis* samples. 1, monotropein; 2, deacetyl asperulosidic acid; 3, asperulosidic acid; 4, asperuloside; 5, rubiadin-1-methyl ether; 6, rubiadin; IS, jaceosidin. Analyte numbering in the test is the same as in this figure.

To achieve good resolution and short analysis time of the selected compounds, several UPLC analytical parameters were optimized, including mobile phase, column type, and temperature, and flow rate of mobile phase. Taking the polarity of anthraquinone and iridoid glycosides into consideration, an Xbridge BEH C_18_ column (150 mm × 4.6 mm, 5 μm; Waters Corp., Milford, MA, USA) was selected for their separation, which provided smoother baseline and superior resolution of the analytes as compared to other chromatographic columns. Then, several organic solvents and aqueous buffer were investigated to minimize peak width and maximize signal intensity. Acetonitrile with stronger elution capability satisfies the requirement of faster elution of analytes and shorter analysis time. Formic acid was selected as the aqueous solvent additive at the concentration of 0.3% over acetic acid. Therefore, the optimal mobile phase, consisting of acetonitrile and water (0.3% formic acid), was finally employed. Furthermore, the effects of column temperature and flow rate were also investigated, and a column temperature of 40 °C and a flow rate of 0.4 mL/min were found to produce the optimal results (Figure 2).

For optimizing the MS/MS parameters, individual analyte solutions (1.0 μg/mL) were directly infused into the mass spectrometer under the positive and negative ionization scan mode. Under the selected electrospray ionization (ESI) condition, all six analytes and IS showed higher sensitivity in the negative ionization mode. Consequently, deprotonated molecules [M − H]^−^ were selected as precursor ions. To obtain the maximum response of precursor and product ions, the parameters for fragment voltage and collision energy were further optimized. Other parameters—such as ESI source, desolvation temperature, and flow rate of the desolvation gas and cone gas—were also optimized. All the MRM transitions and parameters applied in the study are shown in Table 1.

### 2.2. Optimization of Sample Preparation

**Figure 3:**
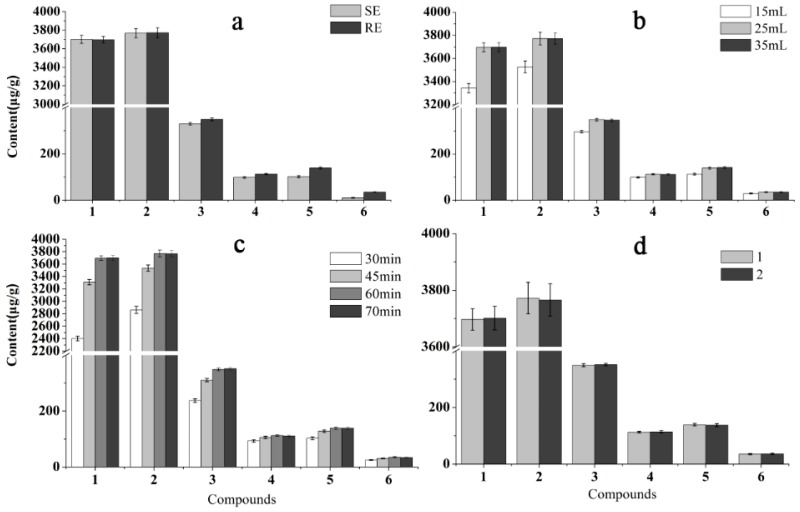
Effects of (**a**) method (SE, sonication extraction; RE, refluxing extraction); (**b**) solvent volume; (**c**) extraction time; and (**d**) extraction repetition on the extraction efficiency of target analytes in S13 *M. officinalis* sample from Hainan province.

Sample preparation is a critical step for developing an accurate and reliable LC-MS/MS quantitative analysis of multiple components. To optimize the preparation of sample solution, the extraction method (reflux and ultrasonication), extraction time (30, 45, 60, and 75 min), solvent volume (15, 25, and 35 mL), and extraction repetition (1 and 2 times) were investigated using the sample S13 collected from Hainan province of China. Regarding the extraction method, heat refluxing extraction showed better extraction yield, especially for rubiadin-1-methyl ether and rubiadin (Figure 3). Hence, refluxing extraction was selected as the extraction approach in this study. For the volume of extraction solvent, 25 mL of methanol was found to be more effective for the samples, as it provided the highest yields of all six ingredients. According to the peak area of analytes, 60 min was selected as the extraction time. Furthermore, extraction repetition (1 and 2 times) was compared and the results indicated that repetition did not show a significant impact on extraction efficiency. As a result, the optimal sample preparation method was summarized as the extraction of 1.0 g sample with 25 mL of methanol by refluxing for 60 min. 

### 2.3. Method Validation

Method validation was performed by evaluating the linearity, limit of detection (LOD), limit of quantification (LOQ), precision, repeatability, stability, and recovery. All results are listed in Table 2. The calibration curves of six target compounds exhibited relatively wide concentration ranges, with correlation coefficients higher than 0.9930. For all ingredients, the LODs ranged from 0.87 to 9.19 ng/mL, and the LOQs from 2.60 to 27.57 ng/mL. The inter- and inter-day precisions of the investigated components exhibited an RSD of less than 2.80% and 4.21%, respectively. The average recoveries of these compounds were in the range of 95.32–99.86% with RSD values between 1.75% and 4.23% (Table 3), indicating good reliability and accuracy of the proposed method. In addition, all analytes exhibited good stability and repeatability, with RSDs for the peak area of less than 5.0%.

### 2.4. Sample Analysis

The validated UPLC-QqQ-MS/MS method was applied to simultaneously quantify the six compounds in 17 batches of *M. officinalis* samples collected from different regions in China. In LC-MS, especially in liquid chromatography coupled to electrospray mass spectrometry, the ion suppression/enhancement effects from matrix can significantly reduce or enhance the analyte response. Appropriate internal standard (IS) can overcome ion suppression/enhancement effects. Therefore, IS was used to minimize variation between samples. The target components were identified based on comparison of retention time, precursor, and product ions obtained from MS/MS analysis of the standard compounds. Representative chromatograms of MRM scans of the standards and sample (S13) are shown in Figure 2. Quantitative analysis results are summarized in Table 4. These results indicated that there were significant differences in the contents of monotropein, deacetyl asperulosidic acid, asperulosidic acid, asperuloside, rubiadin-1-methyl ether, and rubiadin among the 17 samples. Monotropein and deacetyl asperulosidic acid were found to be the most abundant in all samples, varying from 1016.25 to 3696.90 μg/g (µg per g of dry weight) and from 817.64 to 3767.48 μg/g, respectively. Similar results have also been reported previously [16]. Asperulosidic acid and asperuloside were relatively abundant and their contents were in the ranges of 33.05–348.87 μg/g and 1.13–112.64 μg/g, respectively. However, the two compounds were not detected in three samples (S3, S5, and S17). Rubiadin-1-methyl ether and rubiadin were detected in all samples but in variable amounts.

In China, *M. officinalis* is mainly distributed in Southern China. Hainan island (18°10′–20°10′ N latitude and 108°37′–111°03′ E longitude) is the only tropical zone of China, and the climate is different from other regions. The content of some compounds (anthraquinones) in Hainan samples being higher than samples from other regions may correlate with the climate. Similar finding has been reported [23]. However, Shi et al. found that the content of five anthraquinones (1-methoxy-2-hydroxy anthraquinone, 1,2-dimethoxy-3-hydroxy anthraquinone, rubiadin-1-methylether, 1,3-dihydroxy-2-methoxy anthraquinone, rubiadin) in Guangxi samples were highest in all samples (except Hainan) [14]. Furthermore, harvest time is also another major factor affecting the content of monotropein in *M. officinalis* [24]. In fact, there is no successful report on relationship between content of compounds in *M. officinalis* and environmental factors, geography, or harvesting time. Fortunately, the proposed UPLC-MS/MS method is suitable for determination of the target analytes in *M. officinalis.*

### 2.5. UPLC-Q/TOF-MS Identification

To date, more than 30 anthraquinones and 7 iridoids have been isolated as the main effective components in the root of *M. officinalis*. These components share the same basic skeleton structure with different substituting groups. Among these compounds, there are seven pairs of isomers in anthraquinones and one pair of isomer (monotropein and deacetyl asperulosidic acid) in iridoid glycosides. Negative ESI ionization mode was found to be more sensitive than the positive ionization mode for detecting iridoid glycosides and anthraquinones [25,26]. Therefore, the target compositions were identified by UPLC-ESI-Q/TOF-MS in negative ionization mode. In the negative ESI experiments, the deprotonated molecule [M − H]^−^ was detected in the MS/MS spectra of all the target analytes within 5.0 ppm (Table 5). In addition, comparison of the retention time and MS/MS spectra information was achieved by using the reference compounds. Due to similar structures of the target components—for example, between deacetyl asperulosidic acid and monotropein—the fragmentation pathways of iridoid glycosides and anthraquinones in *M. officinalis* were examined.

**Figure 4:**
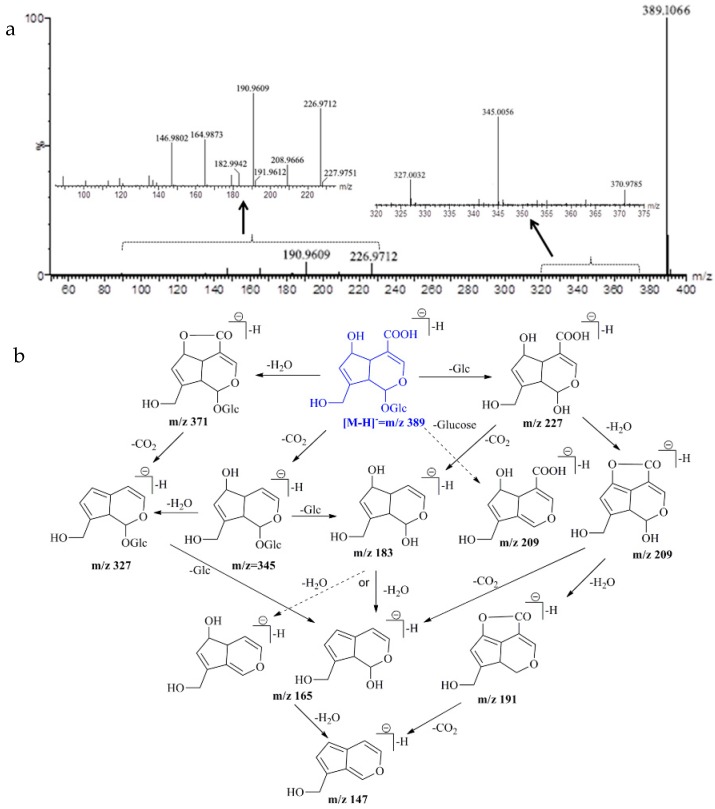
MS/MS spectra (**a**) and the proposed fragmentation pathway (**b**) of deacetyl asperulosidic acid.

The MS/MS spectra and fragmentation pathway of deacetyl asperulosidic acid is shown in Figure 4. The main and typical losses of this compound are H_2_O (18 Da), CO_2_ (44 Da), and glucose (Glc) unit (162 Da). Furthermore, deacetyl asperulosidic acid formed a product ion at *m*/*z* 208.96, due to the loss of glucoside (180 Da) from the deprotonated molecule [M − H]^−^ or the loss of one molecule of H_2_O from *m*/*z* 226.97 [M − H − Glc]^−^. The product ions at *m*/*z* 164.98 and *m*/*z* 190.96 were attributed to the loss of one molecule CO_2_ and H_2_O from *m*/*z* 226.97. This is also supported by previous findings of deacetyl asperulosidic acid in this plant and in *Hedyotis diffusa* [27,28].

**Figure 5:**
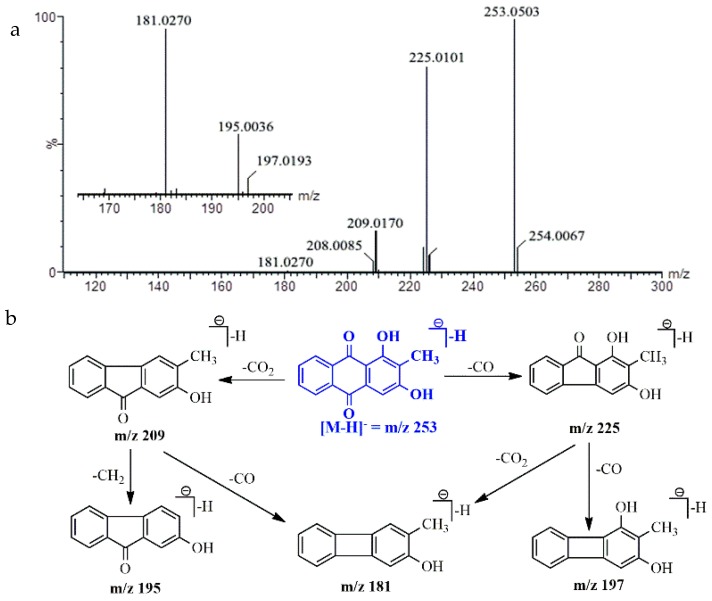
MS/MS spectra (**a**) and the proposed fragmentation pathway (**b**) of rubiadin.

For rubiadin, the product ions at *m*/*z* 225.01 and *m*/*z* 197.01 were formed by the neutral losses of two -CO (28 Da) of the deprotonated molecule [M − H]^−^ at *m*/*z* 253.00. The fragment ion at *m*/*z* 209.01 was formed by the elimination of 44 Da of [M − H]^−^ *m*/*z* 253.00, which corresponded to the loss of one molecule of CO_2_. In addition, the fragment ions *m*/*z* 181.02 and *m*/*z* 195.00 were occurred by the loss of CO (28 Da) and CH_2_ (14 Da) from product ion at *m*/*z* 209.01, respectively. The proposed fragmentation pathway of rubiadin (Figure 5) was in accordance with the previous reports [29,30].

## 3. Materials and Methods

### 3.1. Chemicals, Reagents, and Samples

HPLC-grade acetonitrile, methanol, and formic acid were purchased from Thermo Fisher Scientific (Fisher, Fair Lawn, NJ, USA). Analytical grade methanol and ethyl acetate were supplied by the Beijing Chemical Works (Beijing, China). Water used in the experiment was generated by a Milli-Q water purification system (Millipore, Milford, MA, USA). Reference standards of monotropein, deacetyl asperulosidic acid, asperulosidic acid, and asperuloside were purchased from Shanghai Tauto Bio-Technology Co., LTD. (Shanghai, China). Rubiadin-1-methyl ether, rubiadin, and jaceosidin (internal standard, IS) were obtained from Chengdu Chroma-Biotechnology Co., Ltd. (Chengdu, China). The purities of all standards were above 98.0%. The chemical structures of these reference standards are shown in Figure 1.

The roots (17 batches) of *Morinda officinalis* were collected in December of 2016 from Guangdong, Hainan, Guangxi, and Fujian provinces of China. The samples were identified by Prof. Meihua Yang (Institute of Medicinal Plant Development, Chinese Academy of Medical Sciences & Peking Union Medical College, Beijing, China).

### 3.2. UPLC-QqQ-MS/MS

Chromatographic separation was performed on a UPLC system (Acquity H-Class, Corp., Milford, MA, USA) containing a binary solvent manager, a column manager, and a sample manager. The samples were analyzed on an Xbridge BEH C18 column (150 mm × 4.6 mm, 5 μm; Waters Corp., Milford, MA, USA) and the column temperature was set at 40 °C. The mobile phase consisted of acetonitrile (A) and 0.3% formic acid aqueous solution (B) to enable a gradient elution following the steps: 0 min, 7% A; 5 min, 10% A; 8 min, 45% A; 13 min, 60% A; 22 min, 90%; 25 min 90% A; 30 min, 7% A; finally, the mobile phase was returned to, and remained in, the initial condition for 5 min to re-equilibrate the system before the next injection. The flow rate was 0.4 mL/min and the injection volume was 5 μL. The UPLC system was equipped with a Waters triple quadrupole mass spectrometer (Xevo TQ-D, Waters Corp., Milford, MA, USA) with an electrospray ionization (ESI) source. Analytes were detected and quantified by MRM in negative ionization mode with argon as collision gas. The capillary voltage was set at 2.5 kV. Nitrogen was used as desolvation gas at a flow rate of 650 L/h at a temperature of 350 °C. The flow rate of the cone gas was 150 L/h and the source temperature was 150 °C. Cone voltage and collision energy were individually optimized by direct infusion of analytes and IS into the MS system. MS/MS parameters of the six investigated compounds and IS are listed in Table 1. For LC-MS/MS analysis, data acquisition and handling and instrument control were performed using MassLynx software (Waters Corp., Milford, MA, USA).

### 3.3. UPLC-Q/TOF-MS/MS

The above-described UPLC conditions were used for UPLC-QqQ-MS/MS. A quadrupole time-of-flight mass spectrometer (Xevo G2-XS, Waters Corp., Manchester, UK) equipped with an ESI source was coupled to the UPLC system. Detection was implemented in the MSE centroid mode over a mass range of 500–1200 Da with a scan rate of 10 per second. The analyzer sensitivity mode was used. Leukine encephalin was infused as lockspray via a reference probe for in-run mass correction. Capillary voltage was set at 2.5 kV for ESI. Desolvation gas (nitrogen) was delivered at 600 L/h and 400 °C. Flow rate of cone gas was set to 50 L/h and source temperature was set to 125 °C. Collision energy was ramped in the high energy function from 30 to 50 eV using argon as collision gas. MassLynx software (Waters Corp.) was used for post-acquisition analysis.

### 3.4. Sample Preparation

An aliquot of 1.0 g of *M. officinalis* was extracted by refluxing with 25 mL of methanol for 1 h. The extracted solution was cooled to room temperature and made up to the original weight with methanol. The obtained solution was filtered and subsequently centrifuged for 15 min at 12,000 rpm. Then, 0.5 mL of IS (1.6 μg/mL) was add to 0.5 mL of the supernatant fluid and the mixture was vortexed for 30 s. A 5 μL aliquot was introduced into the UPLC-MS/MS system for analysis. All samples were determined in triplicate.

### 3.5. Standard Solution Preparation

Appropriate amounts of monotropein, deacetyl asperulosidic acid, asperulosidic acid, asperuloside, rubiadin-1-methyl ether, and rubiadin were separately weighed and dissolved in methanol to get the stock solutions. Then, the six stock solutions were mixed and diluted with methanol to prepare a final mixed standard solution containing 159.3 μg/mL of monotropein, 192 μg/mL of deacetylasperulosidic acid, 42 μg/mL of asperulosidic acid, 75 μg/mL of asperuloside, 43.5 μg/mL of rubiadin-1-methyl ether, and 45 μg/mL of rubiadin, respectively. A series of working solutions of these ingredients were obtained by diluting mixed standard solution with methanol at the appropriate concentrations. A specific quantity of jaceosidin was dissolved in methanol to produce the IS solution with a concentration of 0.8 μg/mL. All the solutions were stored at 4 °C before use.

### 3.6. Method Validation

#### 3.6.1. Linearity and Limit of Quantification

The calibration curves of nine concentration levels of each standard were constructed by plotting the peak area ratio of the analyte to IS versus their concentration. Limits of detection (LODs) and quantification (LOQs) were determined at a signal-to-noise ratio (S/N) of about 3 and 10, respectively.

#### 3.6.2. Precision, Repeatability, and Stability

Precision was determined by replicated analyses (*n* = 5) of standard samples within one day (intra-day variation) and on three consecutive days (inter-day variation). Then, the RSD value of the peak area for each analyte was computed. To confirm repeatability, six different sample solutions prepared from the same sample were analyzed, and variations were expressed by RSD. For stability investigation, one of the sample solutions (S13) was stored at room temperature (about 25 °C) and analyzed at 0, 2, 4, 8, 16, and 24 h, respectively.

#### 3.6.3. Recovery

Recovery test was carried out for the accuracy of the established method. The test was carried out by adding known amount of the six standards at low (80% of the known amount), medium (the same as the known amount), and high (120% of the known amount) levels. The spiked samples were then extracted, processed, and quantified according to the abovementioned methods. The average recovery was calculated by the following formula: recovery (%) = (found amount − original amount)/spiked amount × 100.

## 4. Conclusions

For the first time, a rapid, sensitive, and convenient UPLC-ESI-QqQ-MS/MS method was developed and validated for the simultaneous determination of four iridoid glycosides (monotropein, deacetyl asperulosidic acid, asperulosidic acid, and asperuloside) and two anthraquinones (rubiadin-1-methyl ether and rubiadin) in *M. officinalis*. The developed method offers the advantages of high sensitivity and simple sample preparation. It was successfully applied to simultaneously quantify the six bioactive components in 17 batches of *M. officinalis* samples collected from different regions of China. Results have shown that monotropein and deacetyl asperulosidic acid were the abundant constituents in *M. officinalis*. The two main iridoid glycosides were chosen as the marker compounds for the quality assessment of *M. officinalis*. In addition, the proposed fragmentation behaviors of the analytes may provide a reference for screening iridoid glycosides and anthraquinones in *M. officinalis* due to similarity in their skeleton and fragment groups.

## Figures and Tables

**Table 1:** MS parameters of the target analytes and jaceosidin.

Analytes	Retention Time (min)	Parent (*m*/*z*)	Daughter (*m*/*z*) (Q/I) ^a^	Cone Voltage (V)	Collision Energy (eV)
Monotropein	4.03	388.98	146.95/190.95	47	20/25
Deacetyl asperulosidic acid	4.98	389.12	118.93/164.95	45	24/21
Asperulosidic acid	10.96	431.05	251.03/165.01	48	22/20
Asperuloside	11.41	413.03	146.95/190.94	51	18/23
Rubiadin-1-methyl ether	17.42	267.02	223.99/251.95	50	25/25
Rubiadin	20.29	252.97	224.01/209.96	50	30/26
Jaceosidin (IS)	13.73	328.98	313.95/298.96	50	22/22

^a^ Q: transitions for quantification; I: transitions for identification.

**Table 2:** Calibration curves, test range, limit of detection (LOD), limit of quantification (LOQ), precision, and repeatability for the six analytes.

Analytes	Calibration Curves	R^2^	Linear Range (μg/mL)	Precision (RSD, %)		LOQ ng/mL	LOD ng/mL	Repeatability (RSD, %, *n* = 6)
				Intra-Day	Inter-Day			
Monotropein	*Y* = 0.0492*X* + 0.0072	0.9981	0.015–79.65	1.71	3.80	13.87	4.20	2.89
Deacetyl asperulosidic acid	*Y* = 0.0281*X* + 0.001	0.9990	0.030–96.00	2.80	3.83	26.63	8.07	3.92
Asperulosidic acid	*Y* = 0.0252*X* + 0.0018	0.9956	0.042–21.00	1.12	2.56	27.57	9.19	2.54
Asperuloside	*Y* = 0.1235*X* + 0.0073	0.9987	0.018–37.50	2.71	2.74	11.06	3.35	3.17
Rubiadin-1-methyl ether	*Y* = 1.1937 *X* + 0.2136	0.9930	0.008–21.75	2.50	4.21	6.74	2.04	4.02
Rubiadin	*Y* = 0.587*X* + 0.0233	0.9949	0.003–22.50	0.75	2.72	2.60	0.87	2.35

**Table 3:** Recoveries of the six compounds.

Analytes	Sample (g)	Origin (μg)	Spiked (μg)	Found (μg)	Mean Recovery (%) (RSD, %)
Monotropein	0.5	1848.45	1480	3304.33	98.37 (3.23)
			1850	3680.69	99.04 (2.15)
			2220	4059.13	99.58 (3.06)
Deacetyl asperulosidic acid	0.5	1883.74	1500	3351.19	97.83 (4.07)
			1880	3731.03	98.26 (2.38)
			2260	4113.01	98.64 (1.95)
Asperulosidic acid	0.5	174.43	140	310.70	97.33 (2.84)
			175	346.00	98.04 (3.37)
			210	384.14	99.86 (3.69)
Asperuloside	0.5	56.32	45	100.61	98.41 (4.23)
			55	110.13	97.83 (3.94)
			67.5	122.63	98.23 (2.78)
Rubiadin-1-methyl ether	0.5	69.53	55	122.58	96.47 (2.94)
			70	136.56	95.77 (2.62)
			84	149.59	95.32 (3.15)
Rubiadin	0.5	17.62	14	31.27	97.48 (1.75)
			17.5	34.63	97.17 (2.77)
			21	37.88	96.48 (3.28)

**Table 4:** Quantitative analytical results of M. officinalis (μg/g, *n* = 3).

No.	Origins	1	2	3	4	5	6
S1	Guangdong	2497.58	2766.00	246.94	33.95	113.49	11.15
S2	Guangdong	2125.89	2179.01	171.42	8.44	184.22	21.64
S3	Guangdong	1861.98	1771.73	83.22	ND	116.76	7.95
S4	Guangdong	2141.90	2162.22	175.05	5.98	107.61	15.67
S5	Guangdong	1016.25	817.64	ND	ND	42.93	3.80
S6	Guangdong	1590.15	1510.59	93.76	7.38	57.04	19.31
S7	Guangdong	1540.68	1593.63	46.14	1.09	146.63	17.58
S8	Guangdong	1513.27	1458.78	67.97	6.04	143.97	208.70
S9	Guangdong	1827.98	1695.86	86.50	3.08	87.69	12.79
S10	Hainan	2310.95	1990.72	91.30	15.19	81.43	28.17
S11	Hainan	1907.49	1697.96	151.21	15.03	118.81	38.88
S12	Hainan	3020.44	3308.44	234.72	19.95	65.85	7.01
S13	Hainan	3696.90	3767.48	348.87	112.64	139.05	35.25
S14	Guangxi	2046.66	1838.41	50.22	1.13	116.43	10.84
S15	Guangxi	1521.74	1784.73	116.21	11.25	167.85	24.15
S16	Fujian	1940.38	1805.78	33.05	2.07	172.22	20.89
S17	Fujian	1266.58	1199.37	ND	ND	123.04	11.40

ND: not detected.

**Table 5:** Mass data of the six analytes from *M. officinalis* by UPLC-Q/TOF-MS.

Analyte	Molecular Formula	Theoretical Mass (Da)	Measured Mass (Da)	Error (ppm)	Fragment Ions (ESI^−^, *m*/*z*)
Monotropein	C1_6_H_22_O_11_	389.1084 [M − H]^−^	389.1066 [M − H]^−^	−4.62	226.9712, 190.9609, 164.9873, 146.9802
Deacetyl asperulosidic acid	C_16_H_22_O_11_	389.1084 [M − H]^−^	389.1072 [M − H]^−^	−3.08	146.9305, 165.0189, 227.0082, 190.9921
Asperulosidic acid	C_18_H_24_O_12_	431.1190 [M − H]^−^	431.1184 [M − H]^−^	−1.39	146.9305, 165.0189, 251.0062, 119.0033
Asperuloside	C_18_H_22_O_11_	413.1084 [M − H]^−^	413.1092 [M − H]^−^	1.94	146.9926, 190.9722, 233.0963, 369.1387
Rubiadin-1-methyl ether	C_16_H_12_O_4_	267.0657 [M − H]^−^	267.0653 [M − H]^−^	1.50	224.0036, 252.9935
Rubiadin	C_15_H_10_O_4_	253.0501 [M − H]^−^	253.0503 [M − H]^−^	0.79	225.0101, 209.0170, 181.0270
